# Physician Workforce Disparities and Patient Care: A Narrative Review

**DOI:** 10.1089/heq.2019.0040

**Published:** 2019-07-01

**Authors:** Julie K. Silver, Allison C. Bean, Chloe Slocum, Julie A. Poorman, Adam Tenforde, Cheri A. Blauwet, Rebecca A. Kirch, Ranna Parekh, Hermioni L. Amonoo, Ross Zafonte, David Osterbur

**Affiliations:** ^1^ Department of Physical Medicine and Rehabilitation, Harvard Medical School, Spaulding Rehabilitation Network, Massachusetts General Hospital, and Brigham and Women's Hospital, Boston, Massachusetts.; ^2^ Department of Rehabilitation and Human Performance, Icahn School of Medicine at Mount Sinai, New York, New York.; ^3^ Department of Physical Medicine and Rehabilitation, Harvard Medical School, Spaulding Rehabilitation Network, and Massachusetts General Hospital, Boston, Massachusetts.; ^4^ Department of Physical Medicine and Rehabilitation, Harvard Medical School and Spaulding Rehabilitation Network, Boston, Massachusetts.; ^5^ Department of Physical Medicine and Rehabilitation, Harvard Medical School, Brigham and Women's Hospital, and Spaulding Rehabilitation Network, Boston, Massachusetts.; ^6^ National Patient Advocate Foundation, Washington, District of Columbia.; ^7^ Department of Psychiatry, Massachusetts General Hospital, Boston, Massachusetts.; ^8^ American Psychiatric Association, Washington, District of Columbia.; ^9^ Department of Psychiatry, Harvard Medical School and Brigham and Women's Hospital, Boston, Massachusetts.; ^10^ Department of Psychosocial Oncology and Palliative Care, Dana-Farber Cancer Institute, Boston, Massachusetts.; ^11^ Countway Library of Medicine, Harvard Medical School, Boston, Massachusetts.

**Keywords:** women in medicine, women physicians, Black physicians, Hispanic physicians, physician burnout

## Abstract

**Background:** Ensuring the strength of the physician workforce is essential to optimizing patient care. Challenges that undermine the profession include inequities in advancement, high levels of burnout, reduced career duration, and elevated risk for mental health problems, including suicide. This narrative review explores whether physicians within four subpopulations represented in the workforce at levels lower than predicted from their numbers in the general population—women, racial and ethnic minorities in medicine, sexual and gender minorities, and people with disabilities—are at elevated risk for these problems, and if present, how these problems might be addressed to support patient care. In essence, the underlying question this narrative review explores is as follows: *Do physician workforce disparities affect patient care?* While numerous articles and high-profile reports have examined the relationship between *workforce diversity* and patient care, to our knowledge, this is the first review to examine the important relationship between *diversity-related workforce disparities* and patient care.

**Methods:** Five databases (PubMed, the Cochrane Library of Systematic Reviews, EMBASE, Web of Knowledge, and EBSCO Discovery Service) were searched by a librarian. Additional resources were included by authors, as deemed relevant to the investigation.

**Results:** The initial database searches identified 440 potentially relevant articles. Articles were categorized according to subtopics, including (1) underrepresented physicians and support for vulnerable patient populations; (2) factors that could exacerbate the projected physician deficit; (3) methods of addressing disparities among underrepresented physicians to support patient care; or (4) excluded (*n*=155). The authors identified another 220 potentially relevant articles. Of 505 potentially relevant articles, 199 (39.4%) were included in this review.

**Conclusions:** This report demonstrates an important gap in the literature regarding the impact of physician workforce disparities and their effect on patient care. This is a critical public health issue and should be urgently addressed in future research and considered in clinical practice and policy decision-making.

## Introduction

Ensuring the strength of the physician workforce is essential to optimizing patient care. Yet, current projections show there will be a deficit of between 42,600 and 121,300 physicians by 2030 in the United States, with the majority of this deficit localized to primary care providers.^1^ Moreover, “If underserved populations had care utilization patterns similar to populations with fewer access barriers, demand for physicians could rise substantially.”^1^ Even as the demand for health services rises, major problems exist in the physician workforce, including inadequate diversity,^2–5^ decreased recruitment of underrepresented minorities,^6^ decreased recruitment of physicians into nonprimary care specialties (e.g., surgical specialties and other nonmedical specialties such as psychiatry and pathology),^1^ inadequate funding and/or legislative/societal support for funding for physician training,^7,8^ inequities in advancement,^9^ attrition,^10^ high levels of burnout,^11^ an aging physician population,^1^ and elevated suicide risk.^12^

Diversity of the physician workforce has been the subject of numerous reports and studies. Generally, the representation of physicians identifying with four populations is at levels lower than predicted from their numbers in the general population: women, racial and ethnic minorities in medicine, sexual and gender minorities, and people with disabilities. This narrative review explores the role of physicians from these underrepresented groups in patient care, whether physicians from these groups are at elevated risk for problems that could exacerbate the projected physician deficit, and if present, how these problems may be addressed to both support physicians and enhance patient care, especially for the most vulnerable populations.

In essence, the underlying question this narrative explores is as follows: *Do physician workforce disparities affect patient care?* While numerous articles and high-profile reports have examined the relationship between *workforce diversity* and patient care,^2,13^ to our knowledge, this is the first review to examine the important relationship between *diversity-related workforce disparities* and patient care. Our findings are aimed at informing future research, policy, and practice.

## Methods

Five databases (PubMed, the Cochrane Library of Systematic Reviews, EMBASE, Web of Knowledge, and EBSCO Discovery Service) were searched by a medical librarian using appropriate search terms for each database (Table 1) on March 5, 2018. The search was English language and included all reports to date. Additional resources were included by authors as deemed relevant to the investigation.

**Table 1.** Key Search Terms and Results

Database	Search date	Primary search terms	Secondary search terms	Initial results
PubMed	March 5, 2018	“health status disparities”[mesh] or health status disparities[tiab] or “healthcare disparities”[mesh] or healthcare disparities[tiab] or health disparity[tiab] or health disparities[tiab] or “minority health”[mesh] or minority health[tiab]	“physicians, women”[mesh] or “dentists, women”[mesh] or women physicians[tiab] or women dentists[tiab] or women in medicine[tiab] or woman physician[tiab] or woman dentist[tiab] or woman doctor[tiab] or woman dentist[tiab] or female doctor[tiab] or female dentist[tiab] or female doctors[tiab] or female dentists[tiab] or minority physicians[tiab] or minority physician[tiab] or hispanic physicians[tiab] or african physician[tiab] or african physicians[tiab] or african american physician[tiab] or african american physicians[tiab] or black physician[tiab] or black physicians[tiab] or native american physicians[tiab]	50
PubMed	March 5, 2018	“practice patterns, physicians”[mesh] or practice patterns[tiab]	“physicians, women”[mesh] or “dentists, women”[mesh] or women physicians[tiab] or women dentists[tiab] or women in medicine[tiab] or woman physician[tiab] or woman dentist[tiab] or woman doctor[tiab] or woman dentist[tiab] or female doctor[tiab] or female dentist[tiab] or female doctors[tiab] or female dentists[tiab] or minority physicians[tiab] or minority physician[tiab] or hispanic physicians[tiab] or african physician[tiab] or african physicians[tiab] or african american physician[tiab] or african american physicians[tiab] or black physician[tiab] or black physicians[tiab] or native american physicians[tiab]	302
Cochrane Library	March 5, 2018	“healthcare disparities”	“physicians, women”	1
EMBASE	March 5, 2018	“health disparity”/exp or “health disparity” or “health disparities” or “minority health” or “health status disparity”’ or “health status disparities” or “health status disparity” or “healthcare disparities” or “healthcare disparity”	“female physician”/exp or “female physician” or “female physicians” or “woman physician” or “women physicians” or “minority physician” or “black physician” or “black physicians” or “black doctor” or “black doctors” or “african american physician” or “african american physicians” or “hispanic doctor” or “hispanic doctors” or “hispanic physician” or “hispanic physicians” or “female dentist” or “female dentists”’ or “woman dentist” or “women dentists” and ([embase]/lim not [embase]/lim and [medline]/lim)	9
EMBASE	March 5, 2018	“clinical practice”/exp or “clinical practice”	“female physician”/exp or “female physician” or “female physicians” or “woman physician” or “women physicians” or “minority physician” or “black physician” or “black physicians” or “black doctor” or “black doctors” or “african american physician” or “african american physicians” or “hispanic doctor” or “hispanic doctors” or “hispanic physician” or “hispanic physicians” or “female dentist” or “female dentists” or “woman dentist” or “women dentists” and ([embase]/lim not [embase]/lim and [medline]/lim	49
Web of Knowledge	March 5, 2018	“health status disparities” or “healthcare disparities” or “health disparity” or “health disparities” or “minority health”	“women physicians” or “women dentists” or “women in medicine” or “woman physician” or “woman dentist” or “woman doctor” or “woman dentists” or “female doctor” or “female dentist” or “female doctors” or “female dentists” or “minority physicians” or “minority physician” or “hispanic physicians” or “african physician” or “african physicians” or “african american physician” or “african american physicians” or “black physician” or “black physicians” or “native american physicians”	35
Web of Knowledge	March 5, 2018	“physician practice patterns” or “practice patterns”	“women physicians” or “women dentists” or “women in medicine” or “woman physician” or “woman dentist” or “woman doctor” or “woman dentists” or “female doctor” or “female dentist” or “female doctors” or “female dentists” or “minority physicians” or “minority physician” or “hispanic physicians” or “african physician” or “african physicians” or “african american physician” or “african american physicians” or “black physician” or “black physicians” or “native american physicians”	39
EBSCO Discovery Service		women n3 physicians	“healthcare disparities”	Librarian selected 13 from 5140 results

[mesh], search for keywords among; [tiab], search for keywords in title and abstract; /exp, search for keywords among lower levels of the topic hierarchy; and [embase]/lim and [embase]/lim not [embase]/lim, search for articles containing keywords in EMBASE that are not also in Medline; N3, search for keywords within three words of each other.

Institutional review board approval was not required for this study, a review of the literature, as it did not involve participants.

## Results

**FIG. 1.**
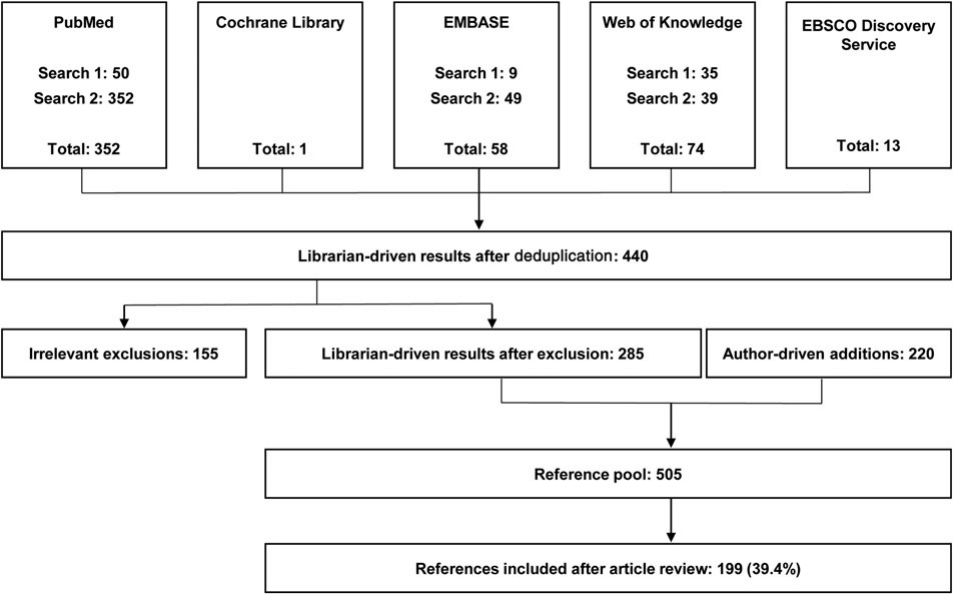
Literature inclusion and exclusion process.

After deduplication, the initial database searches identified 440 potentially relevant articles (Fig. 1). Articles were categorized according to subtopics, including (1) underrepresented physicians and support for vulnerable patient populations; (2) factors that could exacerbate the projected physician deficit; (3) methods of addressing disparities among underrepresented physicians to support patient care; or (4) excluded (*n*=155). After exclusion, 285 potentially relevant articles identified by the librarian-driven search remained. To these, the authors added another 220 potentially relevant articles. Of the 505 potentially relevant articles identified by the medical librarian or authors, 199 (39.4%) were cited because they contained information directly related to the topics included in this review.

Definitions of key abbreviations and terms are listed in Table 2.

**Table 2.** Definitions of Key Abbreviations and Terms

Term	Description
AAMC	Association of American Medical Colleges
Burnout	“A state of physical or emotional exhaustion associated with chronic workplace stress that involves a sense of reduced accomplishment and loss of personal identity”^136^
Cultural competency	“The ability to interact effectively with people of different cultures”^137^ and “be respectful and responsive to the health beliefs and practices—and cultural and linguistic needs—of diverse population groups,”^137^ sometimes also called cultural sensitivity or cultural humility
Effort-reward imbalance	A model developed “to identify health-adverse effects of stressful psychosocial work and employment conditions” that “posits exposure to recurrent experience of failed reciprocity at work ‘high cost/low gain’ increases the risk of incident stress-related disorders”^138^
Explicit bias	Negative or positive attitudes that include “thoughts and feelings that people deliberately think about and can consciously report about”^139^
Gender discrimination	Discrimination based on a person's gender^103^
Gender harassment	The most prevalent type of sexual harassment and constitutes “a broad range of verbal and nonverbal behaviors not aimed at sexual cooperation but that convey insulting, hostile, and degrading attitudes about members of one gender,” including sexist hostility and crude harassment^103^
Implicit bias	“Thoughts and feelings that often exist outside of conscious awareness, and thus are difficult to consciously acknowledge and control”^139^
Intersectionality	“The acknowledgment that within groups of people with a common identity, whether it be gender, sexuality, religion, race, or one of the many other defining aspects of identity, there exist intragroup differences and that individuals may share and experience multiple identities simultaneously”^140^
LGBTQ+	Sexual and gender minority groups
Work-life balance	The “comfortable state of equilibrium achieved between an employee's primary priorities of their employment and their private lifestyle,” including time for family, personal relationships, hobbies, and potential responsibilities as a parent and/or caregiver^141^
People with disabilities	Individuals living with “any condition of the body or mind (impairment) that makes it more difficult for the person with the condition to do certain activities (activity limitation) and interact with the world around them (participation restrictions),” such as impairments in hearing, vision, cognition, mobility, social relationships, communication, and/or self-care^142^
Sexual harassment	“Unwelcome sexual advances, requests for sexual favors, and other verbal or physical conduct of a sexual nature constitute sexual harassment when this conduct explicitly or implicitly affects an individual's employment, unreasonably interferes with an individual's work performance, or creates an intimidating, hostile, or offensive work environment”^103,143^
Technical standards	“A statement by a medical school of the (1) essential academic and nonacademic abilities, attributes, and characteristics in the areas of intellectual conceptual, integrative, and quantitative abilities; (2) observational skills; (3) physical abilities; (4) motor functioning; (5) emotional stability; (6) behavioral and social skills; and (7) ethics and professionalism that a medical school applicant or enrolled medical student must possess or be able to acquire, with or without reasonable accommodation, in order to be admitted to, be retained in, and graduate from that school's medical educational program”^144^
Triple aim	A framework developed by the Institute for Healthcare Improvement that describes an approach to optimizing health system performance through simultaneous pursuit of improvement in patients' experience of care (including quality and satisfaction), population health, and reduction in the per capita cost of health care^145^
URM	Underrepresented in medicine; defined by the AAMC as “those racial and ethnic populations that are underrepresented in the medical profession relative to their numbers in the general population”^25^ and was used before 2003 as the acronym for underrepresented minorities, “which consisted of Blacks, Mexican-Americans, Native Americans (i.e., American Indians, Alaska Natives, and Native Hawaiians), and mainland Puerto Ricans”^25^

## The Role of Underrepresented Physicians in Patient Care

According to a 2018 report from the Association of American Medical Colleges (AAMC) regarding accessibility and inclusion, “When health care providers have life experience that more closely matches the experiences of their patients, patients tend to be more satisfied with their care and to adhere to medical advice. This effect has been seen in studies addressing racial, ethnic, and sexual minority communities when the demographics of health care providers reflect those of underserved populations.”^4^ In addition, diversity has been shown to be good for business, including contributions to improved financial returns, income growth, group thinking, objectivity, and innovation.^14,15^

### Women physicians

In 2017, women accounted for 35.2% (313,808 of 891,770) of the active physician workforce.^16^ The greatest numbers of women (>20,000) were found in family medicine/general practice (45,342), internal medicine (43,770), pediatrics (36,945), and obstetrics and gynecology (23,740). However, the proportion of women among physicians varies dramatically by specialty. Of 44 specialties, a far greater percentage of women than men practice pediatrics (63.3%) and obstetrics and gynecology (57.0%) than orthopedic surgery (5.3%).

Women have been credited with many advancements in the delivery of medical care, particularly in the areas of women's health.^17^ Moreover, women physicians are more likely to care for women^18^ and for patients with complex psychosocial issues, and provide more preventive care and counseling^19^ regardless of the sex of the patient.^20^ Greenwood et al. found that female patients were two to three times more likely to survive a heart attack—the leading cause of death among women—if their emergency room physician was a woman.^21,22^ A recent study of 1.5 million Medicare patients also found hospital mortality and readmission rates were lower for patients treated by women hospitalists than those treated by men,^23^ and women have been found to be less likely to be sued for malpractice.^24^

### Physicians from racial and ethnic minority groups

Since 2004, the AAMC has referred to physicians from some racial and ethnic minority groups as being underrepresented in medicine (URM). While this flexible definition allows for adjustment due to changing physician workforce demographics, populations generally included in this group are “Blacks, Mexican-Americans, Native Americans (i.e., American Indians, Alaska Natives, and Native Hawaiians), and mainland Puerto Ricans.”^25^

Although assessing access to care, quality of the services delivered, patient outcomes, and patient satisfaction with care are highly complex; minority patients are more likely to choose a URM physician and are more satisfied with their care when it is provided by a URM physician.^2,26,27^ Moreover, URM physicians are more likely to practice primary care^28^ and to care for minority and vulnerable patients, including those from communities with lower socioeconomic status.^2,29–33^ In one study, URM physicians were noted as caring for “53.5% of minority and 70.4% of non-English-speaking patients,” and were more likely to have patients on Medicaid.^34^

Although 18% and 13% of the U.S. population identify as Hispanic and African American, respectively, as of 2014, the AAMC reported that only 4% of physicians come from these groups.^28^ Furthermore, despite calls by the AAMC in 2006 to increase both the number of medical schools and the enrollment of medical students, the percentages of matriculants from minority groups in 2015 remained low: Black or African American at 6.5%; Hispanic, Latino, or Spanish origin at 6.4%, and American Indian at 0.3%.^3^ The underrepresentation of physicians from these groups is concerning, especially because the Hispanic population is expected to increase by 26% by 2030, followed closely by the non-Hispanic others (19%), blacks (11%), and whites (9%).^1^ Moreover, improved access to medical insurance will increase demand for all physicians, including URM physicians.^1^

### Physicians from sexual and gender minority groups

Historically, sexual and gender minority (LGBTQ+) health care professionals played a crucial role in the management of specific health crises such as the HIV/AIDS epidemic, including advocating for and delivering care to individuals affected by HIV.^35^ In 2014, the AAMC released guidelines for medical schools aimed at improving cultural competency in the care of LGBTQ+ patients.^36–38^ However, health care inequities persist and result in poorer health care outcomes than in the general population, including increased risk for depression, anxiety, HIV/AIDS, breast cancer, anal cancer, myocardial infarction, diabetes, and negative impacts from long-term hormone therapy.^39^

Although a recent study revealed that 7.7% of 14,254 matriculated U.S. medical students voluntarily identified as LGBTQ+,^40^ and percentages may be higher due the voluntary nature of disclosure,^41^ information regarding how many physicians self-identify as LGBTQ+ is sparse. Given projected physician workforce shortages, the need for a more diverse workforce, and the increasing number of American patients self-identifying as LGBTQ+, there is a need for more LGBTQ+-competent physicians.^38,42,43^

### Physicians with disabilities

It is estimated that people with disabilities—including impairments affecting hearing, vision, cognition, mobility, self-care, and/or independent living—represent 20–25% of both the U.S. and global adult population.^44^ Despite a growing prevalence of disability, persons with disability represent an underserved and vulnerable patient population due to lack of physical access, low provider awareness, attitudinal barriers, and poor communication, and experience poorer health outcomes as a result.^45–50^

In 2012, it was reported that students with mobility and sensory impairments represented less than 1% of matriculated medical students.^51^ A more recent study indicated that less than 3% of currently matriculated medical students reported having a disability.^52^ However, this cohort also represented an expanded population in which students with attention-deficit/hyperactivity disorder (ADHD), learning disabilities, and psychological disabilities were most represented, followed by those with a chronic health issue, other functional impairment, visual impairment, mobility disability, and hearing impairment.^52^

Although a 2005 report noted that between 2% and 10% of physicians reported a disability,^53^ documented prevalence thereafter could not be located. Historically, research describing the prevalence of disability among medical trainees and practicing professionals has been limited by incomplete data, underreporting, and variable response rates among institutions, and recent analyses suggest that survey methodology may underestimate actual prevalence.^52,54^

#### Physicians with intersectional identities

Physicians with intersectional identities are those belonging simultaneously to more than one minority or underrepresented group (e.g., a Hispanic woman or a black gay man). In 2014, the AAMC reported that among younger Asian, black, African American, Hispanic, and Latino physicians, women represented a greater proportion of the workforce (52%) than men (48%).^28^ Although it may be reasonable to assume that physicians with intersectional identities serve multiple groups of patients either with singular or similar intersectional backgrounds and were likely included in studies of single underrepresented groups (e.g., women physicians), we found no report documenting studies of physicians with specific intersectional identities.

## Physicians from Underrepresented Groups and Risk for Leaving Medicine

Physician burnout is a public health crisis and the problem is growing.^55–59^ “Not only does the individual suffer decreased self-esteem and a sense of failure, but his or her ability to provide care can be diminished, as can the ability to work with staff and colleagues. Absenteeism, lower productivity, and higher turnover, with subsequent disruptions in patient care continuity and patient disenrollment, can occur.”^19^ Burnout has been associated with increased risk to patient safety, poorer quality of care, and dissatisfaction with care.^60^ Physician burnout is also associated with a desire to decrease clinical hours or leave clinical practice altogether,^61–63^ and may exacerbate workforce shortages that ultimately impact patient care.^62^

Burnout is commonly defined as a triad of symptoms that occur as a result of chronic workplace stress: emotional exhaustion, depersonalization (e.g., distancing oneself from the demands of patient care by perceiving patients as impersonal objects), and low sense of personal accomplishment.^64^ The factors that contribute to physician burnout are complex and vary among those affected. Physician well-being may be affected by work-life imbalance, effort-reward imbalance, lack of autonomy, discrimination, poor mentorship, lack of workplace social support, isolation, work flow related to electronic health records, time pressure, and chaotic work environments.^63,65^

Physician burnout may be associated with an increased risk of suicidality,^65^ and it is estimated that approximately one physician dies by suicide each day.^66^ Men physicians are 1.41 times more likely and women physicians are 2.27 times more likely to die by suicide compared to nonphysicians.^67^ Moreover, women physicians are more than two times more likely to report thoughts of suicide than men physicians.^68^ In medical students, one study found that symptoms of burnout seemed to be associated with future suicidal ideation, while suicidal ideation decreased upon recovery from burnout.^56^

### Burnout in women physicians

In 2017, the percentage of women physicians in active practice was reported at 35.2%,^16^ and the percentage of women among medical school matriculants passed the 50% mark for the first time.^69^ Recently, it has been suggested that the rising percentage of women physicians may have a negative impact on the availability of primary health care services because they work fewer hours, spend longer with patients, and see fewer patients per day.^70^ At the same time, women physicians have had better patient outcomes than men.^21–23,71^ However, women experience burnout at higher rates than men.^72,73^ Indeed, a recent study revealed that 42% of more than 15,000 physicians across 29 specialties reported symptoms of burnout, with more women physicians (48%) reporting burnout than men (38%).^72^ Among primary care physicians, women reported burnout nearly twice as much as men.^63^

Importantly, burnout may affect men and women differently. While men report more symptoms of depersonalization, women are more likely to report emotional exhaustion.^61,74,75^ Some studies show that women report similar levels of overall career satisfaction compared to men,^19,76^ but they may be less satisfied with specific aspects of their careers, including time for relationships at work and at home as well as career advancement opportunities, recognition, and salary.^63,76^

Inequities in work-life balance appear to be an important factor in women physicians' burnout. Patients expect women physicians to have a caring communication style and that may contribute to higher patient satisfaction,^77^ but also creates a heavier burden. Because women spend more time with their patients on average,^19,78^ they would likely need to work more hours per day to see the same number of patients as men physicians or bear negative impacts on compensation. One study reported every 5 h worked per week over 40 h increased the odds of burnout by 12–15%.^79^ Moreover, increasing work hours appears to correlate with a higher incidence of work-family/home conflict and lower overall job satisfaction.^80–82^

Women physicians also continue to spend disproportionately more time—8.5 h per week more—on child and elder care as well as housekeeping duties than their spouses or domestic partners,^83^ which may further contribute to higher levels of emotional exhaustion and burnout.^83–85^ This may explain why women physicians work fewer hours than men on average and are more likely to work part time.^19,86,87^

Effort-reward imbalance contributes to burnout in women physicians. A survey of more than 20,000 physicians across 29 specialties revealed that women primary care physicians had incomes 18% lower than men—up from 16% in 2017—and the gap increased to 36% for women specialists.^86^ The pay gap persists for women physicians even after adjustment for number of hours worked, type of work, and specialty.^88^ In academic medicine, the same is true even after adjusting for factors, including age; race/ethnicity; experience; specialty; department; faculty rank; research, teaching, administrative, or clinical focus; and marital or parental status.^89,90^ As a correlate to compensation, women with no debt had similar rates of high emotional exhaustion to men (40.5% vs. 38.4%); however, when debt levels exceeded $200,000, women reported much higher rates of high emotional exhaustion than men (60.0% vs. 49.8%).^91^

Women are also not equitably promoted. Despite representing more than one-third (35.2%) of the physician workforce,^16^ less than 25% of full professors and less than 18% of department chairs or deans are women.^92^ Women physicians do not equitably receive recognition awards,^93–96^ and there are troubling disparities in speaking,^94,95^ publishing,^97,98^ and leadership opportunities.^99,100^ Organizational barriers^101^ and implicit bias^94,102^ are often cited as major contributing factors supporting ongoing disparities for women in medicine.

Gender-based workplace discrimination and sexual harassment are also a persistent issue for women physicians,^103^ and gender discrimination is a predictive factor for burnout. According to a 2018 report from the National Academies of Science, Engineering and Medicine (NASEM), 40–50% of female medical students experience sexual harassment from academic faculty or staff.^103^ The report noted, “When women experience sexual harassment in the workplace, the professional outcomes include declines in job satisfaction; withdrawal from their organization (i.e., distancing themselves from the work either physically or mentally without actually quitting, having thoughts or intentions of leaving their job, and actually leaving their job); declines in organizational commitment (i.e., feeling disillusioned or angry with the organization); increases in job stress; and declines in productivity or performance.”^103^

A recent study in academic faculty found that 70% of women reported perceived gender bias and 30% reported sexual harassment during their careers, compared with 22% and 4% of men, respectively.^104^ With respect to medical specialty, rates of discrimination and sexual harassment experienced by women physicians were three times higher in cardiology (69% vs. 22%) and five times higher in radiology (25% vs. 5%) when compared with their male counterparts.^105,106^ A survey study of a large group of women physician mothers participating in an online community group found that almost 80% reported gender discrimination, maternal discrimination, or both.^79^

Gender discrimination often has been reported in studies with regard to women not being considered during administrative decision-making, with results suggesting that a lack of perceived workplace control and autonomy contribute significantly to career dissatisfaction and burnout in women physicians.^19^ Other reports have detailed more subtle and likely unintentional (implicit),^102^ yet pervasive and detrimental, discrimination, including microaggressions, microinequities, and bias (likely implicit/unconscious) that affect speaker introductions,^107^ letters of recommendation,^108,109^ evaluations,^110^ hiring decisions,^111^ and reviews.^112^

### Burnout in URM physicians

Several studies provide insight into the effect of burnout on URM physicians.^113–115^ Hispanic doctors have reported higher career satisfaction and lower perceived stress than their white peers, while African American doctors reported similar levels of satisfaction and perceived stress.^116^ It has been suggested that some URM physicians may be more resilient due to educational, professional, and cultural challenges faced during their training^113,117^ and/or that URM physicians experience a “greater sense of community need and involvement” in their practice among lower socioeconomic status communities with vulnerable patients.^33^

Interestingly, a recent study of workforce longevity in Mississippi revealed that physicians from minority groups or those who practice in rural and shortage areas were among those with the longest careers.^118^ However, minority students have “cited racial discrimination, racial prejudice, feelings of isolation, and different cultural expectations” as negatively impacting their medical school experience.^113^ Further study is needed to determine how factors contributing to burnout may differ between URM and nonminority physicians, and more study is needed to identify URM physicians at risk for burnout.

There is some evidence that minority patients may prefer racially concordant physicians.^119,120^ A study of African-American patients and physicians found that racially concordant visits were longer and more satisfying to patients,^26^ and recently, African-American men were found to be more likely to participate in preventative services after being seen by a black physician.^121,122^ However, studies have shown that URM physicians may see a greater proportion of individuals with medical and psychosocial complexity and confront more environmental and structural challenges, while trying to provide high-quality care.^123^ Thus, URM physicians in certain clinical settings may experience a more challenging work environment with less physician autonomy and lower overall job satisfaction.^123^

Notably, these factors may contribute to physician burnout, reducing the number of hours worked or attrition from the workforce entirely.^61–63^ Importantly, because URM physicians are more likely to locate or remain in geographical areas with physician shortages and work with vulnerable patient populations,^124^ loss of URM physicians is likely to disproportionately affect minority/vulnerable patients, further increasing health care disparities.

Similar to women, URM physicians are underrepresented in leadership positions in medicine, likely also affecting income. While 26% of white faculty members held the rank of professor, 19% and 17% of Hispanic and African American physicians reached that same rank, respectively.^28^ Wage gaps have been reported for URM physicians,^86,125^ further impacting URM physicians who disproportionately come from low-income families, who lack generational wealth/physician legacy, and/or who have higher educational debt.^126,127^ As URM physicians have been shown to accumulate more debt and yet receive less compensation, they are likely paying a higher proportion of their income toward debt reduction.^126^ Concern exists whether financial factors may be contributing to burnout^126,127^ and deterring students from minority groups, from even entering the field of medicine.^7^

### Burnout in LGBTQ+ physicians

LGBTQ+ physicians have reported being harassed by heterosexual colleagues, denied referrals, and ostracized.^43^ They are often witness to discriminatory behavior toward other LGBTQ+ individuals—patients, partners, and coworkers.^43^ More than 30% of patients indicated they would switch providers if they found out their physician was gay or the clinic/practice employed openly LGBTQ+ providers.^128^ In the one burnout-related study identified, LBGTQ+ students were 2.62 times more likely than heterosexual students to report burnout with depression, and academic, personal, and family stressors were strongly linked to symptoms.^129^ In another study, not directly focused on burnout, LGBTQ+ students were found to be at greater risk than heterosexual peers for factors that are often linked to burnout such as depression, anxiety, low perceived self-health, harassment, and isolation.^130^

### Burnout in physicians with disabilities and intersectional identities

Unfortunately, we were unable to find any study specifically aimed at examining burnout in physicians with disabilities or with intersectional identities, making these important areas for future research. We postulate that physicians with disabilities or other intersectional identity, likely racial/ethnic, were included in studies of burnout in women. However, because intersectional identities were not stipulated in the studies, conclusions regarding burnout in physicians with intersectional identities could not be addressed.

## Supporting Physicians from Underrepresented Groups in the Workforce

The Accreditation Council for Graduate Medical Education (ACGME) recently updated their common program requirements for residency and fellowship programs. Beginning July 2019, “The program's annual evaluation must include an assessment of the program's efforts to recruit and retain a diverse workforce.”^131,132^ The training and support of physicians from underrepresented groups, however, have a complicated history.

Traditionally, the justification for allowances, special programs, or accommodations was, and often still is, based on the presumption that, once trained, these individuals will care for concordant, underserved, or vulnerable patient populations and therefore help to close gaps in health care service disparities—improving the overall health of the nation.^34,133^ Indeed, we found the literature ripe with studies documenting the benefits of patient-physician concordance,^26,134^ often concluding with statements such as, “increasing ethnic diversity among physicians may be the most direct strategy to improve health care experiences for members of ethnic minority groups.”^26^ Kelly-Blake et al. challenged this “message repetition” and explained that this type of thinking may inadvertently limit the full scope of physicians' practices as well as pursuit of research and leadership opportunities.^135^

Physicians from underrepresented groups have been disproportionately involved in the care of minority, underserved, and vulnerable patients. Moreover, workforce disparities contribute to burnout and the likelihood of physicians from underrepresented groups reducing hours or leaving medicine. Thus, we need to consider how we can better support them so that they may help address disparities in the care of minority, underserved, or vulnerable patient populations.

**FIG. 2.**
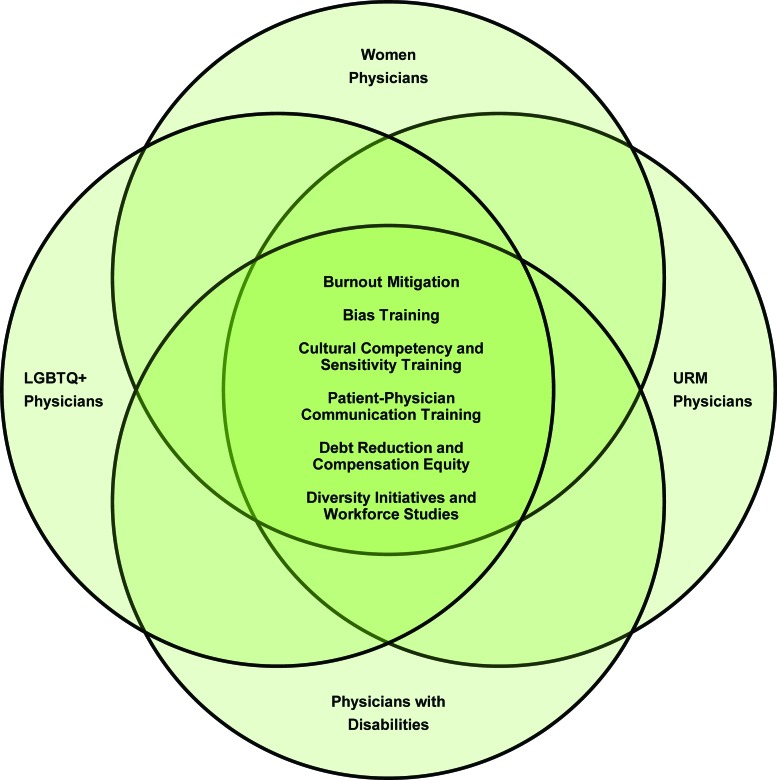
Intersection of support for physicians from underrepresented groups. Color images are available online.

Solutions proposed by researchers and health care leaders to address issues identified in earlier sections are listed in Table 3. Notably, it is possible, although not proven, that some of the proposed solutions might be applicable to more than one of the underrepresented physician groups (Fig. 2), meaning that some single interventions may be capable of impacting multiple target groups, particularly those with intersectional identities.

**Table 3.** Proposed Supports for Underrepresented Physicians

Group	Objective	Targeted interventions proposed
All underrepresented physicians	Burnout mitigation Reduce stress Reduce permanent, temporary, and full-time withdrawals from medical practice Remove burnout as disincentive to medical career and/or specialty choice	Well-being programs^146^ Create and appoint chief wellness officers who implement strategies aimed at the practice environment, teamwork and community building, leadership engagement, compassion for self and colleagues, and support for physicians experiencing distress^147^ Studies aimed at better understanding the variation in burnout and career regret across specialties^57^
	Bias training Improve communication Improve health outcomes Improve patient satisfaction Remove bias as a disincentive to medical career and/or specialty choice	Education regarding how explicit (conscious) or implicit (unconscious) bias as well as the continuum from microinequities to macroinequities or aggressions can impact both professional interactions and patient care^26,148,149^ Completion of the Black-White Implicit Association Test during training to increase awareness of personal implicit bias^150^ Reduction or avoidance of negative comments from higher ranking physicians or negative interactions with patients^150^ Use of equitable language during introductions,^107^ blinded grant applications,^112^ standardized letters of recommendation,^151^ and conscious editing to remove negative or stereotypic language from letters of recommendation^109^ Increased exposure to diversity in educational and workplace settings^48,150^ Education and competencies for physicians-in-training with respect to meeting the needs of LGBTQ+ and other sexual and gender minority groups and effective incorporation of this information into the medical curriculum and clinical experience^152,153^ Creation of an affirming climate and improved accessibility to accommodations for students with disabilities^4^
	Cultural competency and sensitivity training Improve communication Improve patient access to care Accelerate Triple Aim^154^ Remove cultural differences as a disincentive to medical career and/or specialty choice Improve health outcomes	Enactment and/or recommendation of national mandates and guidelines to improve workforce diversity and require cultural competency training^155–162^ Frame cultural competency training in terms of developing understanding of both the patient's and the physician's own cultural backgrounds and unconscious biases^163^ Acknowledge cultural competency as a life-long process, not an end-point, analogous to developing cultural sensitivity or cultural humility^163,164^ Advocate for collaborative relationships that value differing points of view in an effort to improve outcomes^164^ Tailor interactions to patient social, cultural, and communication preferences and needs^156,158^ Increase access to high-quality care services for the medically underserved^165^ Assess institutional readiness to address patient communication and environmental needs^158^ Strengthen the medical research agenda by improving diversity among both researchers and study participants^165^ Expand the pool of medically trained executives ready for health care system and governmental leadership roles^165^
	Patient-physician communication training Improve exchange of information Improve health outcomes Maintain or reduce appointment length Improve scheduling control Improve patient satisfaction Improve reimbursement outcomes	Use patient-centered, conversational communication style consisting of more individualized, reciprocal and supportive responses and notetaking^122,166,167^ Identify patient's preferred language, communication needs, and assistive devices^158^ Improve awareness of patient affective cues^166,168^ Use patient decision aids and navigation^169^ Encourage use and participation of patient companions^170^
	Debt reduction and compensation equity Remove debt as a disincentive to medical career and/or specialty choice, and/or practice location Remove compensation inequity as a disincentive to medical career and/or specialty choice, and/or practice location	Free or reduced medical school tuition^171–173^ Loan repayment, repayment delays, or loan forgiveness^174,175^ School-sponsored financial planning courses and/or access to personal finance experts^175^ State-sponsored financial incentive programs to attract qualified professionals^174^ Transparency in and public reporting of administrative salary information^89^ Accountability and initiatives to combat inequity across specialties and institutions^89,176–178^ Transparency in defining criteria for compensation^179^ Base pay structures on objective criteria^179^ Mitigate implicit bias in compensation decisions, including those regarding salary and bonuses^179^
	Diversity initiatives and workforce studies Improve patient access to care Accelerate Triple Aim^154^	Enhancement of diversity standards in medical and other professional training schools^131,132,174^ Establishment of workforce centers or clearinghouses to monitor data on the supply and demand for specific providers^174^ Evaluate the effectiveness of educational and workforce strategies^174^
Women physicians	Sexual harassment	Develop methodical approaches surveying and combating sexual harassment^180^ Within institutions and organizations, evaluate and address (1) perceived tolerance for sexual harassment, (2) male-dominated workforce, (3) hierarchal power structures, (4) symbolic compliance, and (5) uninformed leadership^103^ Create diverse and respectful environments, improve transparency and accountability, diffuse power, support the targeted individual, and promote strong and diverse leadership^103^
	Gender discrimination	Adoption of systematic guidelines to end gender discrimination and improve the advancement of women in medicine^94,181^ Support rising women physicians through sponsorship,^182^ equitable funding (grant and award),^183^ and equitable collaboration and representation among authors,^97,98^ award recipients,^93,94,183^ faculty, editorial boards,^184^ committee members,^100^ and presidents of medical specialty societies^99^ Improve parental support, including but not limited to “longer paid maternity leave, backup child care, lactation support, and increased schedule flexibility”^79^ Improve support for physicians as family caregivers^19^ Improve control over patient-related decision-making, including but not limited to selection of referral physicians and determination of hospital length of stay^19^ Improve control or influence over work environment such as space and facilities, clinic/office schedule, patient load, and patient characteristics^19^
URM physicians	Access to medical education	Increase public support for historically black medical schools^133^ Increase recruitment of URM physicians through holistic review of applications, conditional acceptance programs, outreach, scholarships, and branch campus locations^185,186^ Increase funding for k-12 education^135^
	Support for and advancement in medical ranks	Recruitment of minority physician faculty^9,135^ Medical specialty society support through education, pipeline programs, clinical care programs, position statements, advocacy, data management, research, and mentorship^187^
LGBTQ+ physicians	Recruitment and workplace culture	Applications allowing declaration of LGBTQ+ status as well as consideration of that status as strengthening applications to medical school^188^ Diversity hiring policies^188^ LGBTQ+ advocates campus-wide, LGBTQ+-friendly training, and LGBTQ+-friendly workplaces (e.g., gender inclusive restrooms)^188^ Partner benefits equivalent to those available to a traditional spouse (e.g., sick leave, maternity leave, and insurance coverage)^188^ Healthy coping strategies, social networks, professional networks, and advocacy groups^189^
	Patient comfort, communication, and outcomes	LGBTQ+-inclusive evidence-based educational materials^188,190^ LGBTQ+-inclusive forms and decision-making tools^188,190^
Physicians with disabilities	Recruitment and workforce culture	Include disability in discussions of diversity^4,191,192^ Increase recruitment^193^ Remove pressure on students and physicians to disclose the full nature of their disability^4^ Improve and standardize medical school technical standards^194^ addressing unclear, inconsistent, and lengthy policies and processes^4^ Define responsibility for accommodations^4,194^ Provide access to appropriate accommodations, personal and professional networks, peer support, and mentorship^4^ Expand study of barriers and accommodations supportive of physicians^194–196^
	Patient comfort, communication, and outcomes	Improve access, provider awareness, and communication, and address attitudinal barriers^45–49,195,197–199^

## Limitations

Although the list of keywords used to search for studies related to the role of physicians from underrepresented groups in medicine seemed inclusive, we found that these keywords were not sufficient to identify all of the studies relevant to this report. We postulate that this discrepancy is due, in part, to inconsistent use of keywords to tag reports applicable to the groups of physicians and patients included in this study. Nevertheless, some sections of this report are more expansive than others because some groups of underrepresented physicians have been studied more (e.g., women physicians) than others (e.g., URM, LGBTQ+, and physicians with disabilities). Moreover, it is also likely that some of the studies included could have addressed intersectionality, but did not. For example, studies of women physicians likely included women physicians who were also URM.

## Conclusion

This report describes an evolving literature that demonstrates the potential impacts of workforce disparities on physicians from underrepresented groups and, in turn, patient care. Despite historical and contemporary challenges, physicians from underrepresented groups—including women, URM, and LGBTQ+, and those with disabilities—are crucial to health care access and quality for the broader U.S. population. As diversity efforts improve, expand, and impact the state of medicine in the U.S., it will be imperative for policymakers, stakeholders, and researchers to consider the unique experiences of physicians from these underrepresented groups when working together to address potential workforce shortages and physician well-being.

Because reasons for inequity may differ between underrepresented groups of physicians, we call on every institution and organization to examine equity in a manner consistent with a six-step data-driven and evidence-based procedure proposed for women in medical specialty societies, which involves identification of inequities, investigation of root causes, implementation of and measurement of outcomes associated with compensatory strategies, and reporting of results to all stakeholders.^94,100^

## Abbreviations Used

AAMC: Association of American Medical Colleges
URM: underrepresented in medicine
